# Evolution of age and length at maturation of Alaskan salmon under size-selective harvest

**DOI:** 10.1111/eva.12123

**Published:** 2013-11-12

**Authors:** Neala W Kendall, Ulf Dieckmann, Mikko Heino, André E Punt, Thomas P Quinn

**Affiliations:** 1 School of Aquatic and Fishery Sciences, University of WashingtonSeattle, WA, USA; 2 International Institute of Applied Systems AnalysisLaxenburg, Austria; 3 Department of Biology, University of BergenBergen, Norway; 4 Institute of Marine ResearchBergen, Norway; † Washington Department of Fish and WildlifeOlympia, WA, USA

**Keywords:** fishery selection, harvest-induced evolution, Oncorhynchus nerka, phenotypic plasticity, probabilistic maturation reaction norms

## Abstract

Spatial and temporal trends and variation in life-history traits, including age and length at maturation, can be influenced by environmental and anthropogenic processes, including size-selective exploitation. Spawning adults in many wild Alaskan sockeye salmon populations have become shorter at a given age over the past half-century, but their age composition has not changed. These fish have been exploited by a gillnet fishery since the late 1800s that has tended to remove the larger fish. Using a rare, long-term dataset, we estimated probabilistic maturation reaction norms (PMRNs) for males and females in nine populations in two basins and correlated these changes with fishery size selection and intensity to determine whether such selection contributed to microevolutionary changes in maturation length. PMRN midpoints decreased in six of nine populations for both sexes, consistent with the harvest. These results support the hypothesis that environmental changes in the ocean (likely from competition) combined with adaptive microevolution (decreased PMRNs) have produced the observed life-history patterns. PMRNs did not decrease in all populations, and we documented differences in magnitude and consistency of size selection and exploitation rates among populations. Incorporating evolutionary considerations and tracking further changes in life-history traits can support continued sustainable exploitation and productivity in these and other exploited natural resources.

## Introduction

Age and size at maturation help to determine an individual's reproductive success and thus its fitness and are also important in the dynamics of populations (Stearns 1992). Locally adapted populations reproducing in different habitats exhibit different patterns of age and length at maturation (e.g., fishes: Beacham 1983; Quinn et al. 2001). Life-history traits, including age and length, at maturation can change rapidly in exploited populations because they are exposed to novel regimes of mortality (Darimont et al. 2009), and exploitation often selectively removes larger individuals (Coltman et al. 2003; Carlson et al. 2007; Kendall and Quinn 2012). The nature of hunting and fishing suggests that this force may lead to microevolutionary trait changes (Policansky 1993; Law 2000; Allendorf et al. 2008; Allendorf and Hard 2009). Such changes take longer to reverse than those associated with phenotypic plasticity alone (Law and Grey 1989).

Few studies have quantified patterns of harvest selection and compare these with associated trait changes over time, especially for multiple stocks of the same species that are differentially harvested. Thus, scientists and managers often lack the ability to correlate harvest with trait changes, understand the mechanisms for these trait changes, and evaluate if and how to modify harvest practices associated with the trait changes. These objectives were accomplished in our study, wherein we estimated average length at age at maturity, age at maturation, and probabilistic maturation reaction norms (PMRNs) of nine heavily exploited sockeye salmon (*Oncorhynchus nerka* Walbaum) populations from two different Alaskan lake systems over five decades. We then compared size-selective fishing mortality with the PMRNs. These results can help scientists and managers address gaps in our understanding and management of harvest-induced selection and evolution and how life-history diversity can be maintained across populations.

PMRNs can help to understand population dynamics and sustainable management by estimating changes in length at age at maturation (Heino et al. 2002a,b; Heino and Dieckmann 2008). PMRNs help to disentangle, to some degree (Heino and Dieckmann 2008; Morita et al. 2009; Uusi-Heikkilä et al. 2011), phenotypic plasticity of life-history traits caused by environmental changes affecting growth and mortality from microevolutionary trait changes associated with size-selective fishing (Olsen et al. 2004; Mollet et al. 2007).

Scientists and managers have recognized that age and length at maturation in Pacific salmon (*Oncorhynchus* sp.) have changed over the past half-century (Ricker 1981; Bigler et al. 1996). These traits are heritable (Carlson and Seamons 2008), and studies demonstrated selection by some fisheries against large size (e.g., Kendall and Quinn 2012). Genetic changes due to size-selective fishing could cause changes in exploited populations (Ricker 1981; Fukuwaka and Morita 2008), but size is influenced by an intricate combination of genetic and environmental factors (Pyper and Peterman 1999).

Sockeye salmon of Bristol Bay, Alaska (see supporting information Fig. S1) are ideal for studying long-term changes in age and length at maturation and shifts in PMRNs as possible microevolutionary changes associated with size-selective fishing. There are large and phenotypically diverse sockeye salmon runs, no stocking from hatcheries, breeding and feeding environments largely unaltered by humans (Hilborn et al. 2003), and size-selective commercial gillnet fisheries that have operated for over 100 years (Kendall et al. 2009; Kendall and Quinn 2012). Sockeye salmon spawn in diverse habitats, and age and length at maturation vary consistently among populations, so they differ in vulnerability to size-selective fishing (Kendall and Quinn 2009).

In this study, we hypothesized that changes in the sockeye salmon life-history traits and PMRN midpoints would be correlated with fishery selectivity patterns, specifically that fish would become shorter at a given age and PMRN midpoints decrease (greater probability of maturing at a shorter length at age) under higher fishing pressure that removes larger than average fish. This would suggest that fisheries-induced evolution is consistent with changes in PMRNs. While a number of other studies have also estimated PMRNs for harvested fish stocks (e.g., Olsen et al. 2004; Mollet et al. 2007; Fukuwaka and Morita 2008; Pardoe et al. 2009) and many have found trends toward maturation at younger ages and/or smaller sizes, our study is rare in two respects.

First, we estimate changes in PMRNs for multiple stocks of the same species. This is important because length and age at maturation and PMRNs may vary among populations (or stocks), due to local adaptation (Taylor 1991) via population-specific selection pressures on the spawning grounds. Because of these differences and variation in fishery selection, PMRNs may evolve in different ways for different populations. Examining these differences can shed light on how selection can act across populations with differing traits, which can inform managers about the potential for life-history evolution in natural populations. Such analyses can also help to understand whether and how life-history diversity can be maintained, supporting the portfolio effect, whereby a diverse ‘portfolio’ of populations and traits among populations increases long-term stability (Schindler et al. 2010), among harvested populations. However, few studies have been able to perform such analyses due to the lack of data or difficulty in differentiating stocks or populations.

Second, our study compares trends in PMRNs with fisheries selection and intensity patterns for the multiple sockeye salmon populations. Again, this is rare given the inability of many studies to accurately estimate size-selective fishing patterns. Such comparisons allow scientists and managers to more confidently associate changes in PMRNs to selective fishing. Previous work by Sharpe and Hendry (2009) related PMRN changes to fishery exploitation rates for multiple fish species. Changes were strongly correlated with fishing intensity, supporting the finding that fishing can play an important role in life-history changes and that such changes may have a genetic basis.

## Methods

### Study site

We studied sockeye salmon populations in two lake systems of Bristol Bay, Alaska. Returning Iliamna Lake sockeye salmon are fished in the Naknek-Kvichak district, whereas Wood River lakes sockeye salmon are fished in the Nushagak district (Fig. S1). Both fisheries have used gillnets since the late 1800s. Fishery size selection has varied over time, but in most years (93% of years for males and 91% for females for the Naknek-Kvichak fishery; 62% of years for males and 89% for females in the Nushagak fishery) since 1963 fish longer than average have been caught, leaving shorter fish to breed (Kendall et al. 2009; Kendall and Quinn 2012).

Data have been collected on two spatial scales since the early 1960s. On a larger scale (all populations together within a fishery), the total catch and escapement (i.e., fish that escape the fishery and can spawn) are estimated, and age, sex, and length (ASL) data have been collected on individual fish for both fisheries by the Alaska Department of Fish and Game as detailed by Kendall and Quinn (2012). On a finer scale (population specific), ASL data have been collected on the Iliamna Lake spawning grounds in most years since the early 1960s and in the Wood River lakes spawning grounds from 1960–1965 and 1990–2009 by the University of Washington Alaska Salmon Program. In general, 110 males and females from each population were sampled, measured for length, and otoliths were collected to age the fish. We analyzed data on five populations from Iliamna Lake and four from the Wood River lakes with the most complete records, spanning the range of spawning sites and fish body sizes and ages (Fig. S1 and Table S1; Quinn et al. 2001). These spawning sites ranged from small streams (<4 m wide) to larger rivers (>75 m wide) with a range of depths and also included beaches. Fish body size and age are correlated with spawning site type, width, and depth (Quinn et al. 2001), with shorter and younger fish spawning in smaller and shallower streams, longer and older fish spawning in larger and deeper rivers, and beach spawners spanning a wider range of sizes and ages.

### Analyses

We first estimated the average length at ocean ages 2 and 3 years of males and females and the proportion of fish of ocean ages 2 and 3 (age composition) in each population over time. We examined temporal differences using linear models. Second, we calculated population-specific PMRNs for ocean age 2 Iliamna Lake sockeye salmon from 41 cohorts since 1960 of sexes and ocean age classes. From the Wood River lakes populations, we estimated ocean age 2 PMRNs over 14 cohorts from 1958–1962 and 1994–2004. This age was chosen because the necessary data were most abundant.

For PMRN estimation, the number and length distribution of immature fish must be compared with those of mature fish at a given age and in a given cohort (Heino et al. 2002a,b). However, length at age distributions of immature salmon are unknown because the fish are only measured at maturity. Therefore, we reconstructed the immature fish length distributions based on those of mature fish following methods used previously (Heino et al. 2002a,b).

Length reconstruction was completed separately for each population and cohort. We back-projected the lengths of ocean age 3 years mature fish measured on the spawning grounds 1 year before they matured, thus estimating immature lengths after fish had spent 2 years in the ocean. Salmon marine growth is not linear; Burgner (1991) reported that the increase in body length was convex over time and that length increased most during the first year at sea. Thus, we back-calculated immature lengths 1 year before maturation (l') for fish in a given cohort (*c*) that matured at a given ocean age (*a*; 3 years in this case).


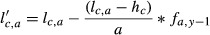
(1)

Here, h_c_ is the average smolt length in a particular cohort for fish leaving Iliamna Lake or the Wood River lakes of a given ocean age (Crawford et al. 1992; Crawford and Fair 2003); *l*_*c*,*a*_ is the mature fish length by cohort and ocean age; and *f*_*a*,*y*-1_ is a growth factor specific to ocean age and represents the proportion of growth associated with the year prior to maturation (from age 2 to 3 years). Growth factors were estimated empirically by Ruggerone et al. (2005) and represent the percent of growth during each year of marine residence (Table S2). Each of the alternate growth factors (Table S2; Lander and Tanonaka 1964; Lander et al. 1966; French et al. 1976) was used in all years in the sensitivity analyses and we also modeled PMRN midpoints using different marine growth factors at different points in the time series. Specifically, we mimicked either a long-term increase in first-year growth conditions (starting with growth factor 1 in 1958–1970, and ending with growth factor 4 in 1991–2004), or a long-term decline in first-year growth conditions (the reverse). It is very difficult to know how temperature trends, changes in fish density in the ocean, and other environmental factors would affect whether sockeye salmon grow more in their first year in the ocean versus in their second and/or third years, and further research is needed on this topic. In these analyses, 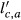
values were re-estimated using each cohort-and age-specific growth factor and used to calculate PMRNs midpoints as described in detail below.

We projected the number of immature fish 1 year before they matured by adjusting the number of mature fish to account for natural mortality, high seas fishing (Myers et al. 1993), and terminal area fishing (Kendall et al. 2009; Kendall and Quinn 2012). Annual cohort-specific offshore mortality rates (*M*_*c*_*yr*^−1^) were estimated as a combination of mortality due to high seas fishing and natural mortality associated with the last year that salmon were in the ocean (between ocean age 2 and 3 years). Furnell and Brett (1986) modeled marine growth and mortality of sockeye salmon and estimated that 90% of the natural mortality at sea occurs in the first 4 months in the ocean. On this basis, we estimated that 10% of the smolts from a given cohort that die in the ocean do so between the end of their first year at sea and their return to spawn following their second or third year in the ocean (Fig. S2).

For Iliamna Lake, but not the Wood River lakes, data on the total number of smolts outmigrating per cohort (*S*_*c*_) were available in many (but not all) years between 1961 and 1998 (e.g., Crawford et al. 1992; Crawford and Fair 2003). Using these data and the total number of adults returning to spawn for each cohort by ocean age (*A*_*c,a*_, a = 2 or 3 years), we first estimated that:


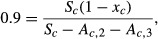
(2)

Where *X*_*c*_ is the cohort-specific survival in their first year in the ocean. Equation 2 can be re-written as:


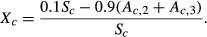
(3)

Therefore, the number of age 1 year immature sockeye salmon (*N*_*c,1*_) alive in the ocean was:


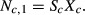
(4)

We then estimated both the cohort-specific number of number of sockeye salmon alive after their second year in the ocean (*N*_*c,2*_; before a fraction of them matured, so immature and mature combined) by multiplying *N*_*c,1*_ by a cohort-specific annual survival rate (*Y*_*c*_) from year 1 to 2 and 2 to 3 in the ocean. We assumed a constant *Y*_*c*_ after age 1 year based on Ricker's (1976) finding of similar values for sockeye salmon in their penultimate and ultimate years in the ocean.


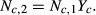
(5)

Because 10% of the mortality occurs after the first year in the ocean:


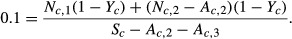
(6)

Substituting equation 4 for *N*_*c,1*_ and equation 5 for *N*_*c,2*_ gives:


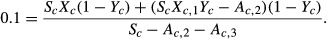
(7)

For each cohort, we solved this equation for *Y*_*c*_ and then estimated annual cohort-specific instantaneous offshore mortality rates for Iliamna Lake sockeye salmon as:


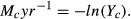
(8)

The values for M_c_ ranged from 0.01 to 1.565 year^−1^ with an average value of 0.588 year^−1^. The large range is likely due to uncertainty in the smolt count estimates; smolt counts are known to be difficult to quantify. In years, for which total smolt counts were not available, we used the average instantaneous M_c_ year^−1^ values estimated in adjacent years where data were available. Annual smolt counts were not available for any years for the Wood River lakes, and thus, we used the average M_c_ estimated for Iliamna Lake sockeye salmon (0.588 year^−1^). A range of offshore instantaneous mortality rates for sockeye salmon that were deemed unbiased and specific to the ultimate year of life in the ocean (0.1 and 0.3* *year^−1^; Ricker 1976) along with larger values (0.5 and 0.8* *year^−1^) were used in the sensitivity analyses. For this analysis, M_c_ yr^−1^ values were re-estimated using each offshore instantaneous mortality rate and then used to calculate PMRNs midpoints as described in detail below.

Inshore fishing mortality rates by the Naknek-Kvichak and Nushagak fishing districts were calculated as the proportion of fish caught (*u*) per year (*y*), by 10 mm length bins (*l*), as in Kendall et al. (2009). These proportions, ranging from 0.005 to 0.98, were calculated for all fish of both sexes.

We then estimated the number of immature individuals (*i*) by length (*l*'), ocean age (*a*; 2 in this case), sex (*s*), cohort (*c*), and spawning population (*p*) one year (*y*) prior to their maturation (for sockeye salmon maturing at ocean age 3) using the number of mature fish (*n*) that were measured on the spawning grounds using equation:


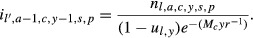
(9)

PMRNs were calculated for sex-cohort-population groupings of ocean age 2 years that had ten or more length at age data points available for both mature and immature fish (so that small samples sizes would not skew the results). The probability of a fish maturing (*o*) was calculated from the individual mature and immature fish data using logistic regression with a binomial error distribution, as maturation is a binary response variable (Heino et al. 2002a,b). We used the generalized linear model (GLM) framework in the program R (R Development Core Team 2011). Different GLMs were used to fit *o* (Table 1) based on length, population, cohort, and sex (e.g., Equation 10).

**Table 1:** Models used to predict maturation of Iliamna Lake and Wood River lakes sockeye salmon, and thus estimate PMRNs, along with their ΔAICc values (the difference between each model's AICc value and that of the model with the lowest value).

	Iliamna Lake		Wood River Lakes	
Variables in model	# parameters	ΔAICc	# parameters	ΔAICc
Length + cohort	43	2554	15	943
Length ^*^ cohort	84	2397	28	901
Length + sex	3	3912	3	5552
Length ^*^ sex	4	3589	4	5339
Length + population	6	4097	5	3565
Length ^*^ population	10	4031	8	3564
Length + cohort + sex	44	1436	16	477
Length + cohort ^*^ sex	85	1350	29	478
Length ^*^ cohort + sex	85	1317	29	442
Length + population + sex	7	2741	6	3270
Length + population ^*^ sex	11	2660	9	3214
Length ^*^ population + sex	11	2681	9	3270
Length ^*^ sex + population	8	2482	7	3153
Length ^*^ sex + cohort	45	1184	17	437
Length + population + cohort	47	1690	18	517
Length + population ^*^ cohort	211	102	57	405
Length ^*^ population + cohort	51	1647	21	456
Length ^*^ cohort + population	88	1544	31	455
Length ^*^ cohort + sex	85	1317	29	442
Length + population + cohort + sex	48	640	19	129
Length + population ^*^ cohort + sex	212	236	58	53
Length + population + cohort ^*^ sex	89	558	32	125
Length ^*^ population + cohort + sex	52	581	22	114
Length ^*^ population + cohort ^*^ sex	93	479	35	106
Length + cohort + population ^*^ sex	52	596	22	113
Length ^*^ cohort + population + sex	89	539	32	85
Length ^*^ cohort + population ^*^ sex	93	486	35	79
Length ^*^ sex + cohort + population	49	412	20	78
Length ^*^ sex + cohort ^*^ population	213	0	59	0


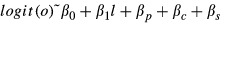
(10)

We also fitted models that included two-way interactions between the predictor variables. We selected the best models using calculated AIC_c_ values. Utilizing the best model, we calculated the length at which the probability of maturing was 50% (*L*_*P50*_) to illustrate the midpoint of the PMRN:


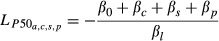
(11)

Temporal variation in *L*_*P50*_ values was evaluated by assessing the significance of the coefficients for the cohort terms in the GLMs and by regressing the predicted *L*_*P50*_ values against cohort. We also examined the significance of the population and sex terms in the GLMs to evaluate differences in *L*_*P50*_ values among populations and between males and females.

We estimated the uncertainty associated with the *L*_*P50*_ values by bootstrapping the original data. For each cohort and population, by sex, and ocean age, we sampled the length data 1000 times with replacement, used these data to recalculate the immature lengths and counts, fitted the ‘best’ model to the generated proportions-by-length and predicted *L*_*P50*_ values.

We calculated two metrics describing the fishing mortality and size selectivity experienced by fish in each population over time using methods detailed in Kendall and Quinn (2009): 1) each population's annual exploitation rate (*V*_*p*,*y*_) and 2) population-specific length-based standardized selection differentials by year (*SSD*_*p*,*y*_). Population-specific exploitation rates and SSDs in most years for the Wood River system populations were previous presented in Kendall and Quinn (2009), but those for the Iliamna Lake populations have not been previously estimated. We examined the relationships between each population's *L*_*P50*_ trends and population-specific fishing exploitation rates and *SSDs*.

## Results

The average lengths at ocean ages 2 and 3 years of male and female sockeye salmon spawning in tributaries of Iliamna Lake and the Wood River lakes have decreased over time (Fig. S3). The slopes of average length of fish for all ocean age-sex-population groups over time were negative, and significantly so for 10 of the 20 Iliamna Lake groups (linear models, *P* < 0.01 required by Šidák correction for multiple comparisons, *F* = 9.8–80.3 for significant groups) and all 16 of the Wood River lakes groups (linear models, *P* < 0.01, *F* = 25.4–228.4). Linear regression slopes of age compositions over time for the various populations included a range of negative and positive values, and no statistically significant trends in age composition were detected for fish of either sex in any Iliamna (linear models, *P* > 0.01, *F* = 0.0–3.9) or Wood River lakes population (linear models, *P* > 0.01, *F* = 0.0–6.4).

**Figure 1:**
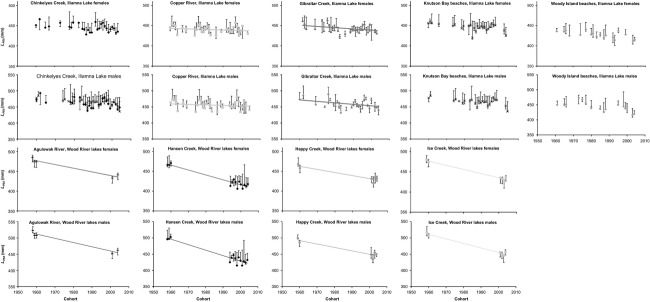
Ocean age 2 sockeye salmon *L*_P50_ values for females and males of Iliamna Lake and Wood River lakes populations. Error bars are 95% CIs estimated from bootstrap analysis. Best-fit lines are for the populations where *L*_P50_ values decreased significantly over time (males and females from Copper River and Gibraltar Creek in Iliamna Lake and males and females from all populations in the Wood River lakes).

The GLMs indicated that length, population, cohort, and sex all affected maturation (Table 1). The GLM *P*-values associated with many cohorts were <0.05 for the best-fit model and the AIC_c_ value of a model not including the cohort term was much larger than the model including it (Table 1), emphasizing variation in PMRNs over time. Linear regression models showed that *L*_*P50*_ values for ocean age 2 fish decreased over time for males and females in all populations. These decreases were statistically significant for both males and females in two of five Iliamna Lake populations (linear models, slope *P* < 0.01, *F* = 9.0–11.6 for significant populations) and for males and females in all four Wood River lakes populations (linear models, slope *P* < 0.01, *F* = 31.5–188.7). Most populations in the best-fit GLM also had corresponding *P*-values <0.05, suggesting significant differences in *L*_*P50*_ values among them, and that of sex was <0.001 for both Iliamna and Wood River lakes, signifying that this term was very important to understand *L*_*P50*_ differences and thus that males and females had different *L*_*P50*_ values. AIC_c_ values of GLM models not including the population or sex term were much larger than models with them (Table 1), emphasizing variation among populations and that female PMRNs were significantly different than those of males. Iliamna Lake *L*_*P50*_ values (determined by linear regression; details above) decreased by 0.1–0.4 mm per cohort for females (Fig. 1A) and 0.2–0.7 mm per cohort for males (Fig. 1B) between the 1960 and 2004 cohorts. Wood River lakes *L*_*P50*_ values declined even more, by 0.8–1.3 mm per cohort for females (Fig. 1C) and 1.1–1.7 mm per cohort for males (Fig. 1D) between the 1958 and 2004 cohorts.

*L*_*P50*_ values changed little across the range of ocean mortality rates used in the sensitivity analysis (Table S3). Even when the ocean mortality rate of 0.8* *year^−1^ was used, there were no differences in the overall conclusions. *L*_*P50*_ values did depend on marine growth factors, decreasing with time for all populations and growth factor combinations by 4.7–45.4 mm for Iliamna Lake populations and 5.4–45.6 mm for Wood River lakes populations. Using growth factor combination 4 (Table S2), we found differences in the statistical significance of *L*_*P50*_ declines for Iliamna Lake populations but not for Wood River lakes populations. Specifically, for Iliamna Lake fish *L*_*P50*_ values still declined for all populations but were significant for only one sex-populations group, whereas originally this was seen for four groups. When growth factor 1 was used in the early years and growth factor 4 in the later years, the decline in the *L*_*P50*_ values was not statistically significant for any population-sex group, but when growth factor 4 was used in the early years and growth factor 1 in the later years, a statistically significant (sharp) decline in *L*_*P50*_ values was seen for all population-sex groups. Thus, the overall patterns of decline were, for most but not all ocean growth models, robust to growth rate variation.

Estimated average standardized selection differentials (*SSDs*) were negative for all Iliamna (one-sided *t*-test, *P* < 0.0001 for each, *t* = −9.5 to −6.7; Fig. S4 and Table S4) and Wood River lakes populations (one-sided *t*-test, *P* < 0.0001, *t* = −5.1 to −4.5). Additionally, linear regressions showed that exploitation rates increased over time for all populations (though only the increase for Gibraltar Creek females was statistically significant at the 0.01 level for Iliamna population-sex groups [*P* = 0.005, *F* = 9.2], while the exploitation rate increases for half of the population-sex groups were significant for the Wood River lakes [*P* < 0.01, *F* = 18–59.8]). Both of these findings were consistent with the overall decreases in the PMRNs across all populations. Exploitation rates were higher for Wood River lakes fish (average = 0.61 for all populations and cohorts vs. Iliamna Lake average = 0.45; *t*-test, *P* < 0.00001, *t* = 7.5; Fig. S4 and Table S4) and *SSDs* were more negative for Wood River lakes fish (average = −0.26 vs. Iliamna Lake average = −0.14; *t*-test, *P* = 0.00003, *t* = −4.4). Consistent with these differences, *L*_*P50*_ values decreased more for Wood River populations than for Iliamna populations.

The Iliamna Lake populations did not vary significantly in estimated exploitation rates or *SSDs* and thus, not surprisingly, these features were not linked to differences in PMRNs. For the Wood River lakes populations, though, size-selective fishing may have influenced changes in PMRNs over time more than overall exploitation. Specifically, we found larger declines in *L*_*P50*_ values for sockeye salmon from Hansen Creek (average of 1.3 mm decline per cohort for females and 1.7 mm for males) than Agulowak River (average of 0.9 mm decline per cohort for females and 1.3 mm for males; Fig. 1). Accordingly, since the early 1960s, *SSDs* were more frequently negative and greater in magnitude for Hansen Creek and other shorter-bodied populations than populations with longer fish (e.g., Agulowak River and Ice Creek; Kendall and Quinn 2009).

## Discussion

All 36 sockeye salmon age-sex-population groups from Iliamna Lake and the Wood River lakes have become shorter at maturity, 26 (72%) statistically significantly so, since the early 1960s. The significant decreases in length at age ranged from 22 to 37 mm for Iliamna Lake populations and 62–106 mm for Wood River lakes populations. However, the proportions maturing after three versus 2 years at sea have not changed significantly in these populations. Morita et al. (2005) concluded that decreases in Pacific salmon length at maturation, concurrent with increases in age, may be adaptive, plastic responses to reduced growth rate. However, our results suggested that factors besides growth, most likely size-selective fishing, have contributed to trait shifts in Bristol Bay sockeye salmon.

We applied the PMRN methodology for the first time to multiple spawning populations of an exploited salmonid stock, estimating PMRNs for sockeye salmon populations using five decades of data from mature fish to back-calculate the number and length of immature fish. *L*_*P50*_ values declined over time for all populations and decreased significantly in 4 of 10 Iliamna Lake sex-population groups and in all 8 Wood River lakes groups. These reductions in *L*_*P50*_ values indicated that the declines in length at age were not only related to changes in growth or mortality over the decades.

Bristol Bay sockeye salmon have experienced heavy but variable size-selective gillnet fisheries since the late 1800s (Kendall et al. 2009; Kendall and Quinn 2012) with significantly negative *SSDs* for all populations (i.e., fish longer than average have been removed, leaving shorter individuals to spawn). The decreases in *L*_*P50*_ values over time are consistent with this size-selective fishing. Our results support the work of Bromaghin et al. (2011), whose individual based model indicated that age and length of harvested western Alaska Chinook salmon were likely to decline with continued harvest.

Decreases in *L*_*P50*_ values were not significant for all Bristol Bay sockeye salmon spawning populations, and this may be due to the large variation in fishery selection over time (Kendall and Quinn 2012). Fewer Iliamna Lake populations, with lower exploitation rates and less size selectivity, showed significant changes in PMRNs than Wood River lakes fish. Overall, our findings support the hypothesis that observed declines in length at maturation of fish of a given age were microevolutionary responses to size-selective exploitation and thus represent fisheries-induced evolution. However, significant changes in PMRNs in some populations were not detected, perhaps due to variation in the size-selective exploitation, the exploitation being less size selective, or lower exploitation rates.

In contrast to the length at age and *L*_*P50*_ patterns, we did not find significant changes in age composition in Iliamna and Wood River lakes sockeye salmon. Decreases in the PMRNs suggest that if growing conditions (related to food availability from production or competition, temperature, or other factors) had remained the same over time, we would have seen the fish maturing at younger ages in recent years. Because such shifts in the age structure have not been realized, concurrent changes in growth and in the PMRN could account for the observed patterns. With overall slower growth in the ocean (Seo et al. 2011; Zavolokin et al. 2012), fewer sockeye salmon would have reached the (lower) PMRNs at younger ages, and thus, age composition did not change. Additional factors may have affected age and length at age at maturation such as environmental conditions including freshwater and sea-surface temperatures (Pyper and Peterman 1999), density of salmon at sea including hatchery fish (Bigler et al. 1996; Pyper and Peterman 1999), and changes in species distributions (Hinch et al. 1995).

The sensitivity analysis showed that our PMRN findings were generally insensitive to the marine mortality rate, but PMRNs varied with the different marine growth factors used to estimate immature length distributions. However, even with the most extreme growth factors applied for each cohort and applying growth factors that varied over time, the slopes for PMRNs still decreased, indicating that the overall conclusions are robust to this factor. Further research to understand Bristol Bay sockeye salmon marine growth, and variation in growth over time, could clarify the *L*_*P50*_ trends. Past studies of Pacific salmon PMRNs directly estimated fish length at certain time periods, and thus growth, by measuring annual growth rings on salmon scales (Morita et al. 2005; Fukuwaka and Morita 2008). This was simply not possible in our study due to the number of fish included and because historical scales were not available for measurement. Thus, we used different ocean growth factors to simulate a variety of growth patterns during a fish's marine residence and also varied these growth factors over time to understand how temporal trends in growth conditions, affecting the growth factors, could impact our results. Uncertainty in our estimates of immature fish growth and our inability to model how growth may have changed over time and reflect such changes in our growth factors (specifically how growth rate variation is reflected in the proportion of growth that a fish experienced during its first, second, and third year in the ocean) are limitations in this study.

The degree to which shifts in PMRNs can indicate microevolution remains somewhat uncertain; the methodology has been criticized for not disentangling genetic and environmental effects on maturation other than through length at age (Kraak 2007; Uusi-Heikkilä et al. 2011), and environmental factors can affect PMRNs directly, not just through growth (Morita et al. 2009). For example, temperature can also directly influence maturation, with increasing temperatures being linked to decreasing age and size at maturation (Tobin and Wright 2011). However, offshore waters of the North Pacific Ocean, in which Bristol Bay sockeye salmon reside during their maturation decision period, is one of the few places where temperatures have decreased slightly since the 1950s (Cane et al. 1997; Mantua 2009), inconsistent with the decreases in size at maturation observed for these fish. PMRNs are not a perfect tool but can help track changes in life-history traits and understand the contribution of harvest to microevolutionary changes, and in this case, the conclusion is broadly supported by the data. The trends we observed are unlikely to have resulted only from a progressive shift in environmental conditions because of the spatial heterogeneity and complex temporal variation in ocean conditions affecting salmon growth and survival over the past decades.

Additionally, recent research has found that differences in growth in Chinook salmon in New Zealand under selection in novel environmental conditions can drive evolutionary changes in life-history traits such as age at maturation rather than evolution of the maturation thresholds defining PMRNs (Kinnison et al. 2011). Such evolutionary forcing is not considered by the PMRN approach because PMRNs tease apart life-history trait changes in maturation correlated with changes in growth (often assumed to be phenotypic plasticity) from those unrelated with growth changes, potentially caused by size-selective fishing (Olsen et al. 2004; Mollet et al. 2007). Thus, we must consider that differences in growth rate over time or among populations could also influence evolutionary age and size at maturation trends in Iliamna and Wood River lakes sockeye salmon populations.

Thus, for these fish, changes in growth have likely interacted with size-selective fishing pressures and resulted in the maturation schedules and age and length compositions seen on the spawning grounds. Both phenotypic plasticity, resulting from changing environmental conditions, and adaptive evolution, due to size-selective fishing and environmental and other forces, can contribute to life-history trait changes (Fukuwaka and Morita 2008), and our study is consistent with the interaction of these effects in shaping age and length at maturation.

Our work supports the findings of Sharpe and Hendry (2009) and points to the importance of considering fisheries-induced evolution as an important mechanism affecting life-history traits in exploited species. Fishery managers should be aware of genetic changes associated with size-selective harvest (Allendorf et al. 2008; Allendorf and Hard 2009) and might use data on changes in age and length at maturation to adjust fishing strategies. For example, managers could reduce exploitation rates or change gear regulations to reduce selectivity (Kendall et al. 2009; Garcia et al. 2012; Kendall and Quinn 2012). Overall, Bristol Bay sockeye salmon stocks are quite healthy (Hilborn et al. 2003; Schindler et al. 2010), but managers should be aware that microevolutionary changes in life-history traits may make these populations less able to respond to future environmental or management changes. Reversing trends toward shorter lengths at age may be difficult, while removing the selective pressure on larger fish may slow or stop the changes in maturation length, selection toward the original genotype in the absence of fishing may be weaker than selection caused by intensive fishing (Fukuwaka and Morita 2008; Enberg et al. 2009).

## Data archiving statement

Data for this study are available from the Alaska Department of Fish and Game and the University of Washington's Alaska Salmon Program (http://fish.washington.edu/research/alaska/).

## Supporting Information

**Figure S1**. Map of the study region (a), located in southwest Alaska. (b) Fish are caught in two fishing districts, Nushagak and Naknek-Kvichak. In our study we assessed (c) four populations spawning in the Wood River lakes and (d) five populations spawning in Iliamna Lake. **Figure S2**. Timeline of a cohort of sockeye salmon from Bristol Bay, Alaska. **Figure S3**. Average length at ocean ages 2 and 3 for male and female sockeye salmon in (a) five Iliamna Lake and (b) four Wood River lakes populations. **Figure S4**. Annual proportions of fish caught and standardized selection differentials (SSDs) for the four Wood River lakes sockeye salmon populations and the five Iliamna Lake populations. **Table S1**. Spawning site width, average mid-eye to fork of tail length of mature ocean age 2 fish, and proportion of sockeye salmon of ocean age 2 between 1962 and 2009 for each population (Quinn et al. 2001). **Table S2**. Marine growth factors used to calculate PMRNs in the sensitivity analysis along with the baseline growth factors (from Lander and Tanonaka 1964; —growth factors 1 and 3; Lander et al. 1966; —growth factor 2; French et al. 1976; —growth factor 4; Ruggerone et al. 2005; — baseline growth factor). **Table S3**. The range of LP50 values estimated for Iliamna Lake and Wood River lakes sockeye salmon in the sensitivity analysis based on different marine mortality values and marine growth factors (listed in Table S2). **Table S4**. The average proportion of fish caught and the average standardized selection differential (SSD) over all years with available data for the four Wood River lakes sockeye salmon populations and the five Iliamna Lake populations.
