# Supplementary material for: Evolution of age and length at maturation of Alaskan salmon under size-selective harvest
Source: Evol Appl. 2013 Nov 12;7(2):313–22. doi: 10.1111/eva.12123 (PMC3927891; doi:10.1111/eva.12123)
Supplement: Supplementary file 1 — Figure S1. Map of the study region (a), located in southwest Alaska. (b) Fish are caught in two fishing districts, Nushagak and Naknek-Kvichak. In our study we assessed (c) four populations spawning in the Wood River lakes and (d) five populations spawning in Iliamna Lake. Figure S2. Timeline of a cohort of sockeye salmon from Bristol Bay, Alaska. Figure S3. Average length at ocean ages 2 and 3 for male and female sockeye salmon in (a) five Iliamna Lake and (b) four Wood River lakes populations. Figure S4. Annual proportions of fish caught and standardized selection differentials (SSDs) for the four Wood River lakes sockeye salmon populations and the five Iliamna Lake populations. Table S1. Spawning site width, average mid-eye to fork of tail length of mature ocean age 2 fish, and proportion of sockeye salmon of ocean age 2 between 1962 and 2009 for each population (Quinn et al. 2001). Table S2. Marine growth factors used to calculate PMRNs in the sensitivity analysis along with the baseline growth factors (from Lander and Tanonaka 1964; —growth factors 1 and 3; Lander et al. 1966; —growth factor 2; French et al. 1976; —growth factor 4; Ruggerone et al. 2005; — baseline growth factor). Table S3. The range of LP50 values estimated for Iliamna Lake and Wood River lakes sockeye salmon in the sensitivity analysis based on different marine mortality values and marine growth factors (listed in Table S2). Table S4. The average proportion of fish caught and the average standardized selection differential (SSD) over all years with available data for the four Wood River lakes sockeye salmon populations and the five Iliamna Lake populations. [file eva0007-0313-sd1.pdf]

Supporting information for:

Evolution of age and length at maturation of Alaskan salmon under size-selective harvest

Neala W. Kendall[1,2*], Ulf Dieckmann[3], Mikko Heino[3,4,5], André E. Punt[1], and Thomas P. Quinn[1]

[1] School of Aquatic and Fishery Sciences, University of Washington, Box 355020, Seattle, WA 98195 USA

[2] Present address: Washington Department of Fish and Wildlife, 600 Capitol Way N., Olympia, WA 98501-1091, USA

[3] International Institute of Applied Systems Analysis, Schlossplatz 1, A-2361 Laxenburg Austria

[4] Department of Biology, University of Bergen, Box 7803, N-5020 Bergen, Norway

[5] Institute of Marine Research, Box 1870 Nordnes, N-5187 Bergen, Norway

[*]*Corresponding author*: neala.kendall@dfw.wa.gov

Figure S1. Map of the study region (a), located in southwest Alaska. (b) Fish are caught in two fishing districts, Nushagak and Naknek-Kvichak. In our study we assessed (c) four populations spawning in the Wood River lakes and (d) five populations spawning in Iliamna Lake.

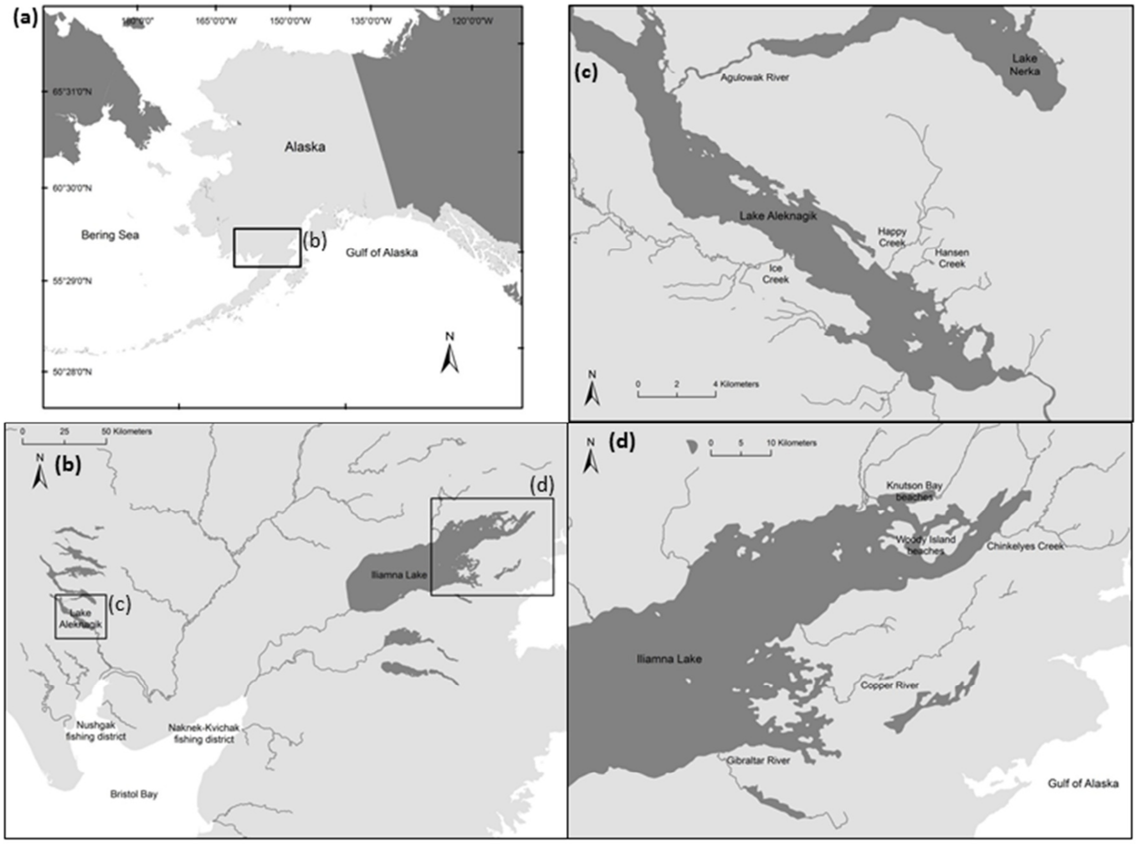

Figure S2. Timeline of a cohort of sockeye salmon from Iliamna Lake, Alaska. Given the number of smolts (S_c) and the number of mature adults of age 2 and 3 years subsequently returning to spawn ($A_{c,a}$, a = 2 or 3 years) along with an estimate of the fraction of total mortality at sea that occurs during the first year (0.9), we first calculated the number of age 1 year fish ($N_{c,1}$) and the cohort-specific survival rate during the first year in the ocean (X_c). With these values, we then calculated the number of age 2 fish ($N_{c,2}$) and an annual ocean mortality value (Y_c) for a fish's second and third year in the ocean. We do not consider fish maturing after 1 and 4 years at sea because they were a very small fraction of the total. Black values were known and grey values were calculated.

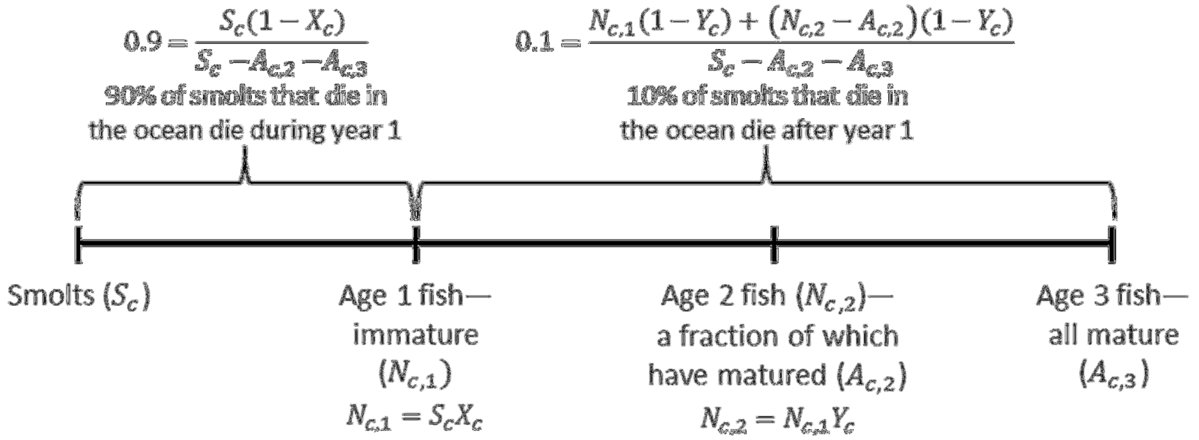

Figure S3. Average length at ocean ages 2 and 3 for male and female sockeye salmon in (a) five Iliamna Lake and (b) four Wood River lakes populations. In (a) trend lines are given for the populations with whose average length decreased significantly over time (Gibraltar Creek ocean age 3 males and females, Gibraltar Creek age 2 males, Woody Island beach ocean age 2 males and females, Woody Island age 3 females, Copper River ocean age 3 males and females, Chinkelyes Creek ocean age 3 males, and Knutson Bay beach ocean age 2 females).

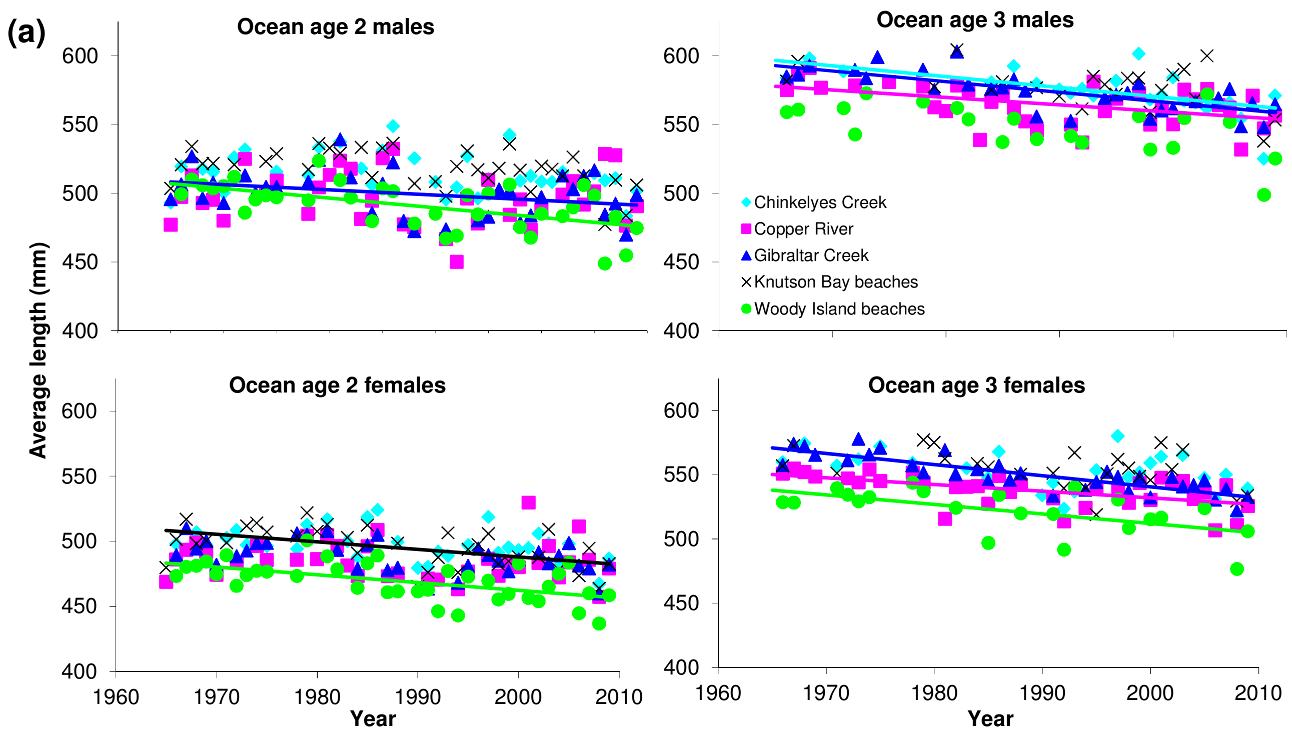

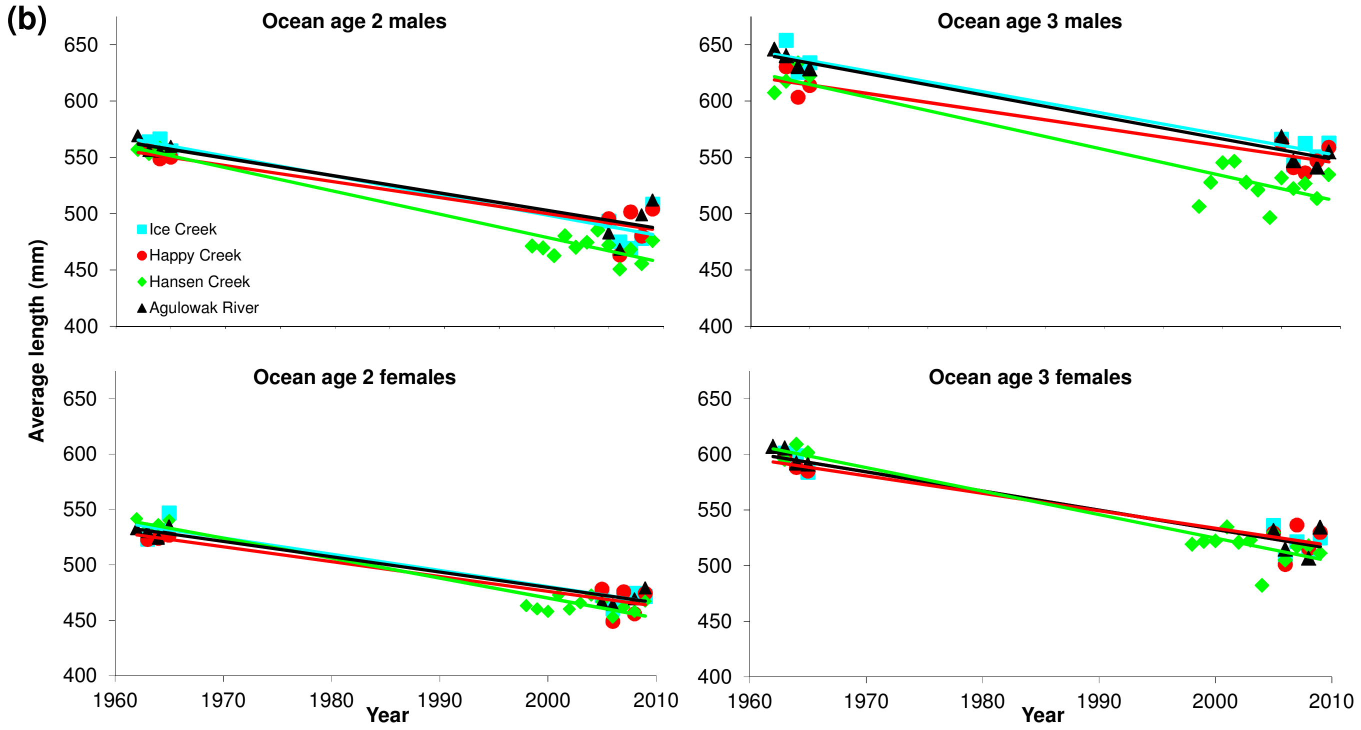
(b)
Ocean age 2 males
Ocean age 3 males
Ocean age 2 females
Ocean age 3 females
Average length (mm)
Year
Ice Creek
Happy Creek
Hansen Creek
Agulowak River
650
600
550
500
450
400
1960
1970
1980
1990
2000
2010

Figure S4. Annual proportions of fish caught and standardized selection differentials (SSDs) for the four Wood River lakes sockeye salmon populations and the five Iliamna Lake populations.

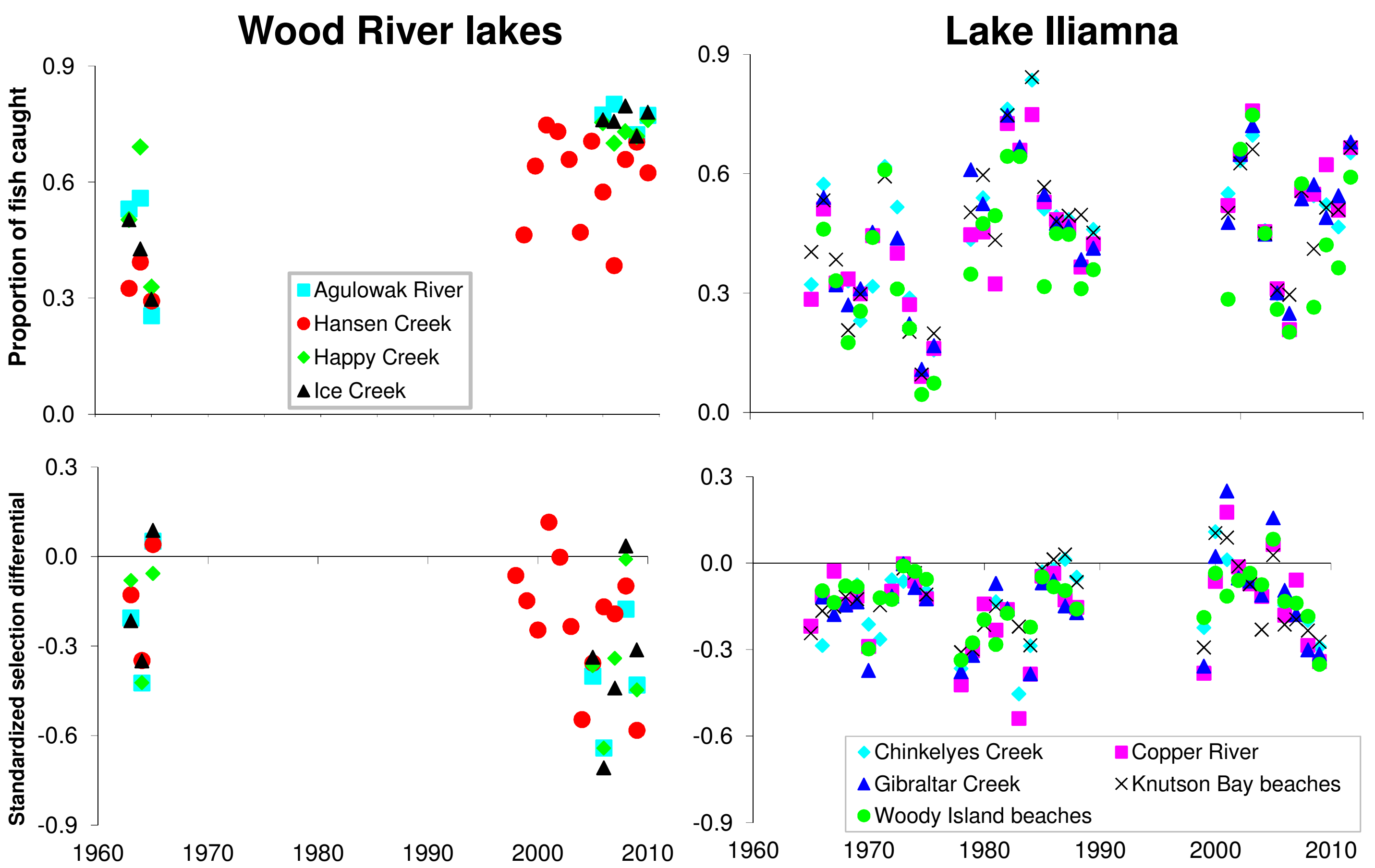

Table S1. Spawning site width, average mid-eye to fork of tail length of mature ocean age 2 fish, and proportion of sockeye salmon of ocean age 2 between 1962 and 2009 for each population (Quinn et al. 2001). Fish body size and age are correlated with spawning site type (small stream, larger river, beach), width, and depth (Quinn et al. 2001).

Lake	Population	Avg. spawning site width (m)	Avg. spawning site depth (cm)	Avg. length ocean age 2 fish (mm)	% ocean age 2 fish
Iliamna	Copper River	21.3	61	490	59
	Gibraltar Creek	28.9	61	495	52
	Chinkelyes Creek	15.2	35.6	504	68
	Woody Island beaches	unconfirmed	155	483	69
	Knutson Bay beaches	unconfirmed	200	507	74
Wood River	Hansen Creek	3.9	9.8	481	77
	Happy Creek	4.7	25.2	501	53
	Ice Creek	15.7	42.4	516	33
	Agulowak River	77.3	46.6	525	29

Table S2. Marine growth factors used to calculate PMRNs in the sensitivity analysis along with the baseline growth factors (from Lander and Tanonaka 1964—growth factors 1 and 3; Lander et al. 1966—growth factor 2; French et al. 1976—growth factor 4; Ruggerone et al. 2005—baseline growth factor).

Growth factors	Year 0–1, ocean age 3	Year 1–2, ocean age 3	Year 2–3, ocean age 3
#1	1.7	0.4	0.9
#2	1.7	0.6	0.7
#3	1.8	0.5	0.7
#4	1.9	0.45	0.65
Baseline	1.075	1	0.925

Table S3. The range of L_{P50} values estimated for Iliamna Lake and Wood River lakes sockeye salmon in the sensitivity analysis based on different marine mortality values and marine growth factors (listed in Table S2).

Lake	Differing factor	Average difference in L_{P50} value (mm)				
		Chinkelyes Creek	Copper River	Gibraltar Creek	Knutson Bay beach	Woody Island beaches
Iliamna		males				
	Ocean mortality rate = 0.1	-0.1	0.0	0.0	0.0	0.0
	Ocean mortality rate = 0.3	-0.7	-1.0	-0.8	-0.7	-0.6
	Ocean mortality rate = 0.5	-1.6	-1.8	-1.7	-1.7	-1.2
	Ocean mortality rate = 0.8	-2.8	-3.1	-2.8	-3.0	-2.2
	Growth factors 1	-5.4	-5.8	-6.0	-5.9	-4.7
	Growth factors 2	-27.1	-27.5	-30.0	-26.9	-22.7
	Growth factors 3	-27.1	-27.5	-30.0	-26.9	-22.7
	Growth factors 4	-32.8	-33.6	-36.7	-32.4	-27.1
		females				
	Ocean mortality rate = 0.1	-0.2	-0.1	-0.1	0.0	-0.1
	Ocean mortality rate = 0.3	-1.0	-1.4	-1.3	-1.1	-1.0
	Ocean mortality rate = 0.5	-2.3	-2.7	-2.5	-2.3	-2.0
	Ocean mortality rate = 0.8	-4.1	-4.5	-4.2	-3.9	-3.7
	Growth factors 1	-6.7	-7.5	-8.3	-7.7	-6.7
	Growth factors 2	-31.3	-33.0	-37.3	-33.0	-26.7
	Growth factors 3	-31.3	-33.0	-37.3	-33.0	-26.7
	Growth factors 4	-37.5	-40.0	-45.4	-39.1	-31.7
		Agulowak River	Hansen Creek	Happy Creek	Ice Creek	
Wood River		males				
	Ocean mortality rate = 0.1	0.0	0.0	0.0	0.0	
	Ocean mortality rate = 0.3	-0.4	-1.0	-2.1	-0.6	
	Ocean mortality rate = 0.5	-1.4	-2.0	-2.8	-1.6	
	Ocean mortality rate = 0.8	-2.3	-3.4	-4.8	-2.7	
	Growth factors 1	-5.8	-6.2	-7.2	-6.7	
	Growth factors 2	-34.8	-28.0	-34.1	-36.2	
	Growth factors 3	-34.8	-28.0	-34.1	-36.2	
	Growth factors 4	-44.4	-33.3	-42.1	-45.6	
		females				
	Ocean mortality rate = 0.1	0.0	0.0	0.0	0.0	
	Ocean mortality rate = 0.3	0.0	-0.3	-0.7	-0.2	
	Ocean mortality rate = 0.5	-1.0	-1.3	-2.0	-1.1	
	Ocean mortality rate = 0.8	-1.5	-2.2	-3.3	-1.8	
	Growth factors 1	-5.4	-5.4	-6.3	-6.0	
	Growth factors 2	-34.7	-26.8	-32.5	-34.7	
	Growth factors 3	-34.7	-26.8	-32.5	-34.7	
	Growth factors 4	-44.0	-31.6	-40.0	-43.6	

Table S4. The average proportion of fish caught and the average standardized selection differential (SSD) over all years with data available for the four Wood River lakes sockeye salmon populations and the five Iliamna Lake populations.

	Males		Females		Male and female average	
**Population**	**Avg. proportion of fish caught**	**Avg. SSD**	**Avg. proportion of fish caught**	**Avg. SSD**	**Avg. proportion of fish caught**	**Avg. SSD**
Agulowak River	0.66	-0.24	0.60	-0.39	0.63	-0.32
Hansen Creek	0.61	-0.19	0.50	-0.20	0.56	-0.20
Happy Creek	0.68	-0.29	0.61	-0.30	0.65	-0.30
Ice Creek	0.68	-0.26	0.58	-0.30	0.63	-0.28
Wood River lakes	0.65	-0.24	0.56	-0.28	0.61	-0.26
Chinkelyes Creek	0.50	-0.13	0.45	-0.15	0.48	-0.14
Copper River	0.48	-0.15	0.43	-0.17	0.45	-0.16
Gibraltar Creek	0.47	-0.12	0.45	-0.16	0.46	-0.14
Knutson Bay beaches	0.48	-0.10	0.46	-0.17	0.47	-0.13
Woody Island beaches	0.43	-0.14	0.36	-0.13	0.39	-0.13
Iliamna Lake	0.47	-0.13	0.43	-0.15	0.45	-0.14

Literature cited:

French, R. , H. T. Bilton, M. Osako, and A. Hartt. 1976. Distribution and origin of sockeye salmon (*Oncorhynchus nerka*) in offshore waters of the North Pacific Ocean.: International North Pacific Fisheries Commission Bulletin.

Lander, A. H., and G. K. Tanonaka. 1964. Marine growth of western Alaskan sockeye slamon (*Oncorhynchus nerka*): International North Pacific Fisheries Commission Bulletin.

Lander, A. H., G. K. Tanonaka, K. N. Thorson, and T. A. Dark. 1966. Ocean mortality and growth: International North Pacific Fisheries Commission Annual Report.

Quinn, T. P., L. Wetzel, S. Bishop, K. Overberg, and D. E. Rogers. 2001. Influence of breeding habitat on bear predation and age at maturity and sexual dimorphism of sockeye salmon populations. Canadian Journal of Zoology **79**:1782-1793.

Ruggerone, G. T., E. Farley, J. L. Nielsen, and P. Hagen. 2005. Seasonal marine growth of Bristol Bay sockeye salmon (*Oncorhynchus nerka*) in relation to competition with Asian pink salmon (*O. gorbuscha*) and the 1977 ocean regime shift. Fishery Bulletin **103**:355-370.
